# Association between psychological resilience and cognitive function in older adults: effect modification by inflammatory status

**DOI:** 10.1007/s11357-021-00406-1

**Published:** 2021-06-29

**Authors:** Sun Jae Jung, Ga Bin Lee, Kristen Nishimi, Lori Chibnik, Karestan C. Koenen, Hyeon Chang Kim

**Affiliations:** 1 Department of Preventive Medicine, Yonsei University College of Medicine, 50-1 Yonsei-ro, Seodaemun-gu, Seoul, 03722 Korea; 2 Department of Public Health, Graduate School of Yonsei University, Seoul, Korea; 3 Department of Epidemiology, Harvard T.H. Chan School of Public Health, Boston, USA; 4 Department of Social Behavioral Science, Harvard T.H. Chan School of Public Health, Boston, USA

**Keywords:** Psychological resilience, Stressful life event, Cognition, MMSE, Inflammatory cytokine, hsCRP, Depression

## Abstract

To examine the association between psychological resilience and cognitive function and investigate the role of acute inflammation as an effect modifier. Total 7535 people from the Cardiovascular and Metabolic Disease Etiology Research Center (CMERC), aged ≥ 50 years and residing in areas near Seoul, South Korea, were included in this cross-sectional analysis. Stressful life events in the past 6 months were gauged by the Life Experience Survey, and current depression symptoms were analyzed with the Beck Depression Inventory-II. Participants were categorized into the following four groups according to their past experience and depression status: reference, resilient, reactive depression, and vulnerable depression. Cognitive function was evaluated using the mini-mental state examination (MMSE). The level of high-sensitivity C-reactive protein (hsCRP) was measured from blood samples. A generalized linear model was used. Upon adjusting for socio-demographic factors, comorbidity, and lifestyle factors, the final model was stratified with the highest quartile of the hsCRP level by sex. Compared to the reference group, the resilient group showed higher MMSE, which was also significant in women (adj-*β* = 0.280, *p*-value < 0.001). Vulnerable depression group showed a significantly lower MMSE (adj-*β* =  − -0.997, *p*-value 0.002), especially in men. This pattern seemed to be limited to the low hsCRP subgroup. We provided evidence from the largest Korean population used to evaluate the association between psychological resilience and cognition, which was more prominent in low inflammatory status. Psychological resilience was associated with a lower likelihood of cognitive deficit in women. This pattern was modulated by inflammatory status.

**Supplementary Information:**

The online version contains supplementary material available at 10.1007/s11357-021-00406-1.

## Introduction

The absolute number of people with dementia is growing rapidly, as 4% of the normal population develop mild cognitive impairment of which 12% progress to developing dementia annually [1]. The decline of cognitive function is known to present in midlife [2]. Various potential factors are known to affect cognitive impairment: age, sex, education, comorbidities, obesity, and lifestyle factors [3]. Additionally, history of depression is a well-known factor which can affect cognitive function at later life. Additionally, there are epidemiologic and biologic evidence to support depression as a neuropsychiatric symptom of vascular dementia (VD) or Alzheimer’s dementia (AD) in early stages [4]. Among 682 individuals with mild cognitive impairment in a cardiovascular health study, about 30% reported concurrent depression [5]. In German patients who had VD or AD, 87% of the participants reported depressive disorder according to the Diagnostic and Statistical Manual of Mental Disorders (DSM-IV) [6]. In a population-based prospective cohort study of Japanese-American men, depressive symptom was associated with dementia by more than seven times compared to the control; however, this finding was limited to the presence of apolipoprotein E ε4 [7]. Especially, when hippocampal volume was measured with structural magnetic resonance imaging (MRI) in people with depression, significant proportion of hippocampal atrophy was found among late-onset depression patients; people with early-onset depression did not show difference in hippocampal volume compared to the control population [8]. These findings may suggest that late-life depression could be a prodrome or subclinical symptom of dementia.

The experience of severe adversities in life has often been mentioned as a contributing factor towards the development of mental illness, including depression. However, researchers observed that certain people stayed mentally healthy regardless of adverse experiences, and thus, constructed the concept of “psychological resilience” [9]. A large proportion of the trauma reported from the community population is related with interpersonal trauma, and women are known to experience more frequent interpersonal trauma across their lifespan, which could also reflect the sex difference in resilience [10]. A prospective study of 276 elderly German population with lower educational level, which was set as the proxy for cognitive reserve (i.e., educational attainment as a mode of resistance to cognitive sequelae), showed that worse cognitive decline was associated with greater amyloid pathology [11]. However, cognitive reserve alone cannot fully explain the resilience, and further comprehensive measurement of psychological resilience is necessary. Moreover, in other epidemiological studies, higher cognitive function was known to be positively correlated to resilience in a relatively younger population [12, 13]. However, there is still a lack of evidence regarding the relationship between psychological resilience and cognitive function in older adult population.

Experiencing trauma or adversity involves responses from the neuroendocrine and immune system, and the differences found in the peripheral immune markers, such as leukocyte interleukin(IL)-6 response after lipopolysaccharide stimulation, correlated resilience to chronic stress in animal models [14]. Pro-inflammatory cytokines, such as C-reactive protein (CRP), are suggested to moderate the process in which psychological stress provokes the development of mental problems and psychological quality of life [15, 16]. Also, cytokine, such as IL-6, is suggested to mediate the process in which psychological stress provokes the development of mental problems, including depression [15]. Furthermore, inflammation is known to provoke the suppression of adult neurogenesis, which could lead to vulnerability of the hippocampus [17]. However, the entire relationship between inflammation, resilience, and cognitive function is not comprehensively understood.

From these results, we hypothesized that psychological resilience will be positively associated with cognitive function, and that inflammation may act as an effect modifier among older adults.

In this study, we aimed to evaluate the association between psychological resilience and global cognitive function by sex using a large population data, and examine the role of inflammation as an effect modifier in the middle-aged population.

## Materials and methods

### Selection of participants

Baseline population data of the Cardiovascular and Metabolic Diseases Etiology Research Center (CMERC) cohort [18], which is an on-going prospective multi-center population study with participants aged 30 to 64 enrolled from 2013 to 2018, was analyzed in this study. Overall, the included population showed similar baseline characteristics of the urban middle class in South Korea [18]. As this cohort primarily aimed to examine early cardio-metabolic risk factors, the initial inclusion was made with community people who were free from myocardial infarct, stroke, and heart failure. A total of 11,964 (4584 men and 7110 women) people provided information about their socio-demographic characteristics, physical status, lifestyle factors, and mental health.

Based on the baseline population data, a follow-up study was conducted in 2019 with 500 randomly selected participants, focusing on their psychological resilience, endocrine measurements, and cardiovascular health. Additionally, from beginning of the SARS-CoV-2 outbreak in 2020, a subgroup of 4060 participants were followed using online questionnaires at three different time points (March 2020, August 2020, and February 2021), and then queried about their mental health response after the pandemic. Recent follow-up survey is currently in progress (April 2021).

**Fig. 1:**
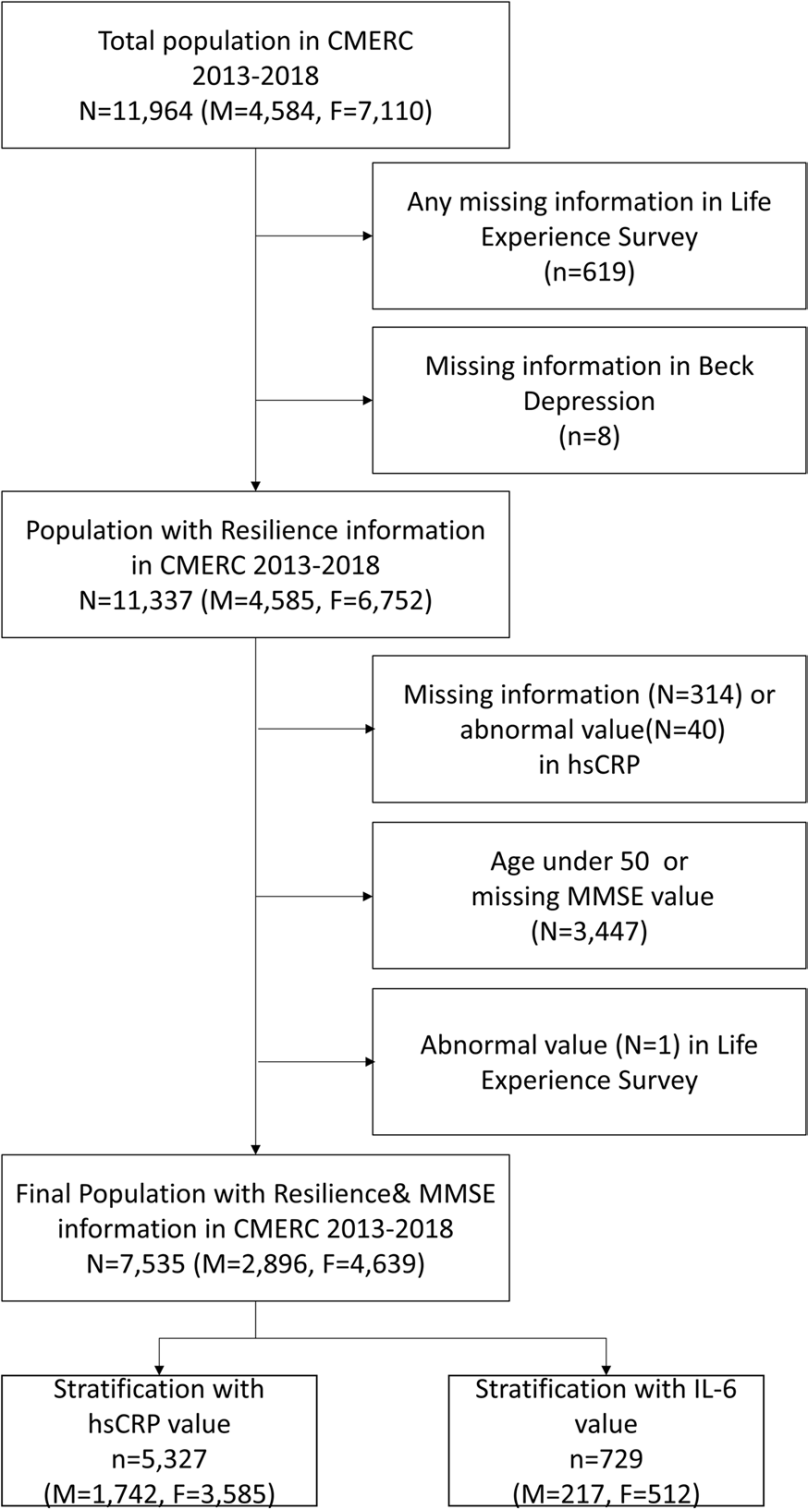
Selection of data from the Cardiovascular and Metabolic Disease Etiology Research Center, 2013–2018

For this study, we excluded participants with any missing information in the Life Experience Survey (LES) (*n* = 619) and missing information in the Beck Depression Inventory-II (BDI-II) (*n* = 8), as these scales were used to define psychological resilience operationally. We additionally excluded participants with missing values or outliers in high-sensitivity C-reactive protein (hsCRP) assessment (*n* = 354) and others with abnormal LES measurement or incomplete mini-mental state examination (MMSE) data (*n* = 11). Among the initially included participants, MMSE was conducted only in participants aged 50 years or above, yielding an age range of 50 to 64 years in the final inclusion, consisting of 7535 participants (2896 were men, and 4639 were women) (Fig. 1). Excluded and included participants did not differ significantly in their body mass index and MMSE scores (Supplementary table 1).

### Measurements of psychological resilience and cognitive function

In the CMERC baseline survey, all items in the questionnaire were queried by trained interviewers. The LES [19], which was also validated in the Korean population, was initially developed to capture the participants’ objective stressful life experiences in the past 6 months and their positive or negative impact perceived subjectively. Initially, the first section of LES contained 47 items, including the death of a close family member, miscarriage, or problems with employers in the workplace. If participants reported having experienced any of these, they were asked to rate its impact from + 3 (extremely positive experience) to − 3 (extremely negative experience), yielding “negative life event” with item scored − 1 to − 3. In our data, we also counted the number of 16 items related to interpersonal adversities. We compared the distribution by sex (i.e., “having conflict with one’s manager,” “having divorced,” or “experiencing a breakup with a friend of the opposite sex.” Full list is presented in Supplementary box 1).

Depressive symptoms in the past 2 weeks were measured with the BDI-II, which was also validated in the Korean population [20]. The BDI-II included 21 items with a Likert scale from 0 to 3, and a higher score reflected severe depressive states. In the Korean population, BDI-II showed high-sensitivity (0.85–0.94) and specificity (0.88–0.98), in which the cutoff of 18 to 22 yielded an area under the curve (AUC) from 93 to 99%. As Beck and Brown suggested [21], we applied the cutoff of 20 and higher to capture moderate-to-severe depression and differentiate the participants into depressed and not depressed groups.

To define psychological resilience operationally, the presence of at least one negative event in the past 6 months and presence of moderate-to-severe depressive symptoms at the time of enrollment were used to create four groups: reference (no negative event; no depression), resilient (with negative events; no depression), reactive depression (with negative events; with depression), and vulnerable depression (no negative event; with depression).

To measure cognitive function and screen cognitive impairment, participants underwent MMSE with trained interviewers at baseline. Out of a maximum score of 30 points of MMSE, the total score with the sum of each item score was acquired. While using the total score as a continuous variable, the outcome variable was created by dichotomizing the total MMSE score to capture the potential cognitive impairment at the clinical level. We applied the different cutoffs of MMSE by each age, sex, and total education years, according to the “MMSE-Dementia Screening (DS)” method proposed by Han et al.[22]; people below -1.5 standard deviations from the distribution of each strata was coded separately.

### Measurement of hsCRP and IL-6

Morning blood samples were collected during the baseline survey, after 8-h fasting, and sent to the single analysis laboratory on the same day. Turbidimetry method with ADIVA 1800 AutoAnalyzer (Siemens Medical Sol., USA) evaluated hsCRP. IL-6 was analyzed using the fluorescence-activated cell sorting analysis and enzyme-linked immunosorbent assay. In our population, we had 5327 (1742 men and 3585 women) people with valid hsCRP and 729 people (217 men and 512 women) with valid IL-6 value. Since there were outliers in hsCRP values, we stratified the association between resilience and cognitive function by hsCRP level, and categorized them into four groups following the distribution. In the same way, categorization by IL-6 level into four groups was also performed. We made comparisons between the highest quartile of hsCRP and IL-6 and other quartiles, which was a common method for comparison of inflammatory biomarkers, including both hsCRP and IL-6 [23]; we also categorized the inflammatory markers with its median values and conducted a sensitivity analysis. Additionally, we conducted a sensitivity analysis using the cutoff of 3.0 mg/L or higher for the hsCRP level, since this cutoff is known to be clinically relevant with other chronic diseases, including cardiovascular conditions [24, 25].

### Covariates

For the demographic variables, final educational level was marked as elementary school, middle school, high school, university or college, and above. For family income, the subjects were asked about their average monthly income, whereas marital status was to be stated as “never married nor cohabited,” “living together with a partner,” “divorced,” “separation by death of a spouse(partner),” and “others.” Comorbidity was queried as “Have you ever been diagnosed with conditions listed by physicians?”; the list contained stroke, transient ischaemic stroke, myocardial infarct, angina, heart failure, chronic renal failure, hypertension, dyslipidaemia, diabetes, thyroid disorders, fatty liver disease, chronic hepatitis, liver cirrhosis, asthma and chronic obstructive pulmonary disease, osteoporosis, arthritis, autoimmune disease, and cancer. Comorbidity was coded as the number of disease conditions. For women, menopausal status and age at menopause were noted. The status of alcohol consumption and smoking was also sought and marked as “never user,” “past user,” and “current user.” We calculated the total time and intensity of physical activities using the World Health Organization guideline [26], and categorized the physically active group as people with at least 150 min of moderate or 75 min of vigorous aerobic activity per week on average.

### Statistical analysis

As previously mentioned, we hypothesized there would be different patterns of association between men and women, and all statistical assessments were made separately for each sex. The association between BDI-II score and MMSE score was tested by multivariable linear regression. Characteristics of the four resilience–depression groups were compared using the analysis of variance and chi-square test. The association between resilience-depression group and MMSE score was evaluated separately for each sex using a generalized linear model compared to the reference group, adjusting for age, study center, education, income, marital status, and comorbidity. Women were additionally adjusted for menopausal status. We examined whether the association between resilience–depression status and MMSE differed according to the inflammatory status after stratification by hsCRP and IL-6 level in both men and women. In every analysis using generalized linear models, the *p*-value was adjusted by Bonferroni correction (0.05/12) for multiple testing. Sensitivity analyses were made with repeating analyses with the bivariate outcome using logistic regression.

All analyses were performed using the SAS, version 9.4 (SAS Institute, Inc., Cary, NC).

### Ethics

The Institutional Review Board of Yonsei University approved the protocol of this study (YUIRB-4–2013-0661), and all participants provided written informed consents. All procedures in this work complied with the ethical standards of the relevant national and institutional committees on human experimentation as per the Helsinki Declaration of 1975 (revised in 2008).

## Results

In our data, women (45%) were more likely to experience interpersonal adversities compared to men (42%), which was statistically significant (*p* = 0.023). Additionally, 7.7% of the women in our population experienced more than three interpersonal adversities in the recent 6 months, compared to 6.2% of men within the same category.

When comparing the number of stressful life events in recent 6 months, we observed a significant difference in the mean number of stressful life events experienced between the resilient group and reactive depression group; the reactive depression group had higher total average number of adverse events compared to the resilient groups in both men and women (Supplementary table 5).

Compared to the reference group, the resilient and reactive depression groups were relatively younger, while the vulnerable depression group was relatively older. The depressed groups appeared to have lower education and lower income, and were less likely partnered. Both groups with depression had a higher proportion of women in the resilient group who were highly educated, married, pre-menopause, and had a higher income. The depression groups showed a higher number of physical comorbidities, including hypertension and diabetes; notably, the vulnerable depression group showed the highest proportion of both hypertension and diabetes out of all groups. The reactive depression group showed a significantly higher proportion of current smokers, and the vulnerable depression group presented a lower proportion of alcohol consumers. Both groups with depression had lesser exercise compared to the other two groups (Table 1).

**Table 1:** Baseline characteristics by resilience status (*N* = 7535)

Participants’ characteristics	Reference^1^ (*n* = 2878: M = 1181, F = 1697)	Resilient^2^ (*n* = 3710: M = 1446, F = 2264)	Reactive depression^3^ (*n* = 735: M = 219, F = 516)	Vulnerable depression^4^ (*n* = 212: M = 50, F = 162)	*p-* value
Men (*n* = 2896)					
Age, mean ± SD	60.26 ± 6.55	59.25 ± 6.51	59.84 ± 6.93	60.32 ± 6.87	0.001
Socio-economic variables					
Education: high school or higher, *n* (%)	544 (46.1)	726 (50.2)	72 (32.9)	14 (28.0)	< 0.001
Highest quartile of yearly household income, *n* (%)	332 (28.1)	374 (25.9)	33 (15.1)	6 (12.0)	< 0.001
Currently married, living together, *n* (%)	1128 (95.5)	1384 (95.7)	193 (88.1)	46 (92.0)	< 0.001
Presence of major comorbidity, *n* (%)	796 (68.5)	1023 (72.0)	164 (76.3)	41 (83.7)	0.080
Hypertension, *n* (%)	683 (57.8)	904 (63.5)	138 (63.0)	35 (70.0)	0.039
Diabetes, *n* (%)	368 (31.2)	409 (28.3)	83 (37.9)	23 (46.0)	0.002
Body mass index (kg/m^2^), mean ± SD	24.99 ± 2.85	25.06 ± 2.86	24.64 ± 3.06	24.49 ± 2.95	0.125
Lifestyle factors, *n* (%)					
Current cigarette smoker, *n* (%)	238 (20.2)	306 (21.1)	76 (34.7)	14 (28.0)	< 0.001
Current alcohol consumer, *n* (%)	864 (73.2)	1080 (74.7)	159 (72.6)	33 (66.0)	0.462
Regular exercise^5^, *n* (%)	495 (41.9)	625 (43.2)	74 (33.8)	16 (32.0)	0.030
Psychiatric assessments					
Beck Depression Inventory^6^ II (range:0–63), mean ± SD	6.06 ± 4.8	8.74 ± 5.21	25.10 ± 5.36	24.12 ± 4.23	< 0.001
Women (*n* = 4639)					
Age, mean ± SD	59.27 ± 6.55	57.73 ± 5.44	58.28 ± 5.42	60.44 ± 6.67	< 0.001
Socio-economic variables					
Education: high school or higher, *n* (%)	382 (22.5)	615 (27.2)	87 (16.9)	26 (16.1)	< 0.001
Highest quartile of yearly household income, *n* (%)	351 (20.7)	513 (22.7)	62 (12.0)	15 (9.3)	< 0.001
Currently married, living together, *n* (%)	1360 (80.1)	1892(83.6)	386 (74.8)	115 (71.0)	< 0.001
Presence of major comorbidity, *n* (%)	1067 (35.7)	1444 (64.3)	372 (73.8)	109 (69.0)	0.001
Hypertension, *n* (%)	755 (44.5)	843 (37.2)	220 (42.6)	77 (47.5)	< 0.001
Diabetes, *n* (%)	327 (19.3)	369 (16.3)	115 (22.3)	120 (25.9)	< 0.001
Body mass index (kg/m^2^), mean ± SD	24.11 ± 3.02	24.04 ± 3.13	24.30 ± 3.31	24.73 ± 3.20	0.023
Lifestyle factors, *n* (%)					
Current cigarette smoker, *n* (%)	21 (1.2)	25 (1.1)	18 (3.5)	5 (3.1)	< 0.001
Current alcohol consumer, *n* (%)	834 (49.2)	1246 (55.0)	282 (54.7)	80 (49.4)	0.002
Regular exercise^5^, *n* (%)	556 (32.8)	724 (32.0)	123 (23.8)	34 (21.0)	< 0.001
Menopause, *n* (%)	1570 (92.5)	2035 (89.9)	486 (94.2)	150 (92.6)	0.002
Psychiatric assessments					
Beck Depression Inventory^6^ II (range:0–63), mean ± SD	7.54 ± 4.94	9.99 ± 4.88	26.40 ± 5.96	25.60 ± 5.78	< 0.001

^1^No negative event in 6 months and no current depressive symptoms (BDI < 20)

^2^Experienced negative events in 6 months but no current depressive symptoms (BDI < 20)

^3^Experienced negative events in 6 months with current depressive symptoms (BDI ≥ 20)

^4^No negative event in 6 months and with current depressive symptoms (BDI ≥ 20)

^5^Defined as having moderate-vigorous physical activity of more than 150 min per week on average

BDI-II was not significantly associated with MMSE (results not shown). In assessing the association between resilience-depression status and cognitive function (Table 2), we observed that resilience was positively associated with better cognition, as gauged by the continuous MMSE score. Especially, women in the resilient group displayed higher cognition (*β* = 0.280, SE = 0.079, *p*-value < 0.001) in the final model as compared to the group with no negative events. Men also exhibited a positive association between resilient state and MMSE score; however, there was no statistical significance (*β* = 0.131, SE = 0.089, *p*-value = 0.138). In contrast, reactive and vulnerable depression groups showed lower cognition in both men and women as compared to the reference group; men with vulnerable depression remained statistically significant for reduced cognitive function even after full adjustment (*β* = –0.997, SE = 0.326, *p*-value = 0.002). The direction of the results did not change according to the dichotomised cognitive outcome defined with MMSE-DS (Supplementary table 2) nor by change in the definition of adversity with two of more negative events in the past 6 months (Supplementary table 2). In the stratified analyses with inflammatory biomarkers, the pattern observed in the main analysis seemed to be limited to the low hsCRP subgroup. (Table 3). We did not find any significant statistical interaction between hsCRP strata and resilience status. However, among people with low hsCRP level, men in the vulnerable depression group also showed significantly lower MMSE scores (adj-*β* = –0.970, *p*-value 0.037), whereas women in the resilient group showed significantly higher MMSE scores (adj-*β* = 0.276, *p*-value 0.004); in contrast, we did not observe any significant result in the high hsCRP group. In women, the results for both reactive and vulnerable depression groups showed different direction of association between MMSE scores by the hsCRP strata, but no result was statistically significant. The results of our sensitivity analyses by categorizing the group with hsCRP with cutoff of 3.0 mg/L showed a similar trend as that of our main analyses (Supplementary table 6) No significant result was found between the resilience status and IL-6 level on MMSE scores (Supplementary table 7).

**Table 2:** Association between resilience status and cognitive function (*N* = 7535)

Resilience status	Mini-mental state examination (continuous)					
	*β*^1^ (SE)	*p* value	*β*^2^ (SE)	*p*-value	*β*^3^ (SE)	*p*-value
In men (*n* = 2896)						
Reference^4^ (*n* = 1181)	0.00 (ref)		0.00 (ref)		0.00 (ref)	
Resilient^5^ (*n* = 1446)	0.156 (0.090)	0.086	0.133 (0.089)	0.134	0.131 (0.089)	0.138
Reactive depression^6^ (*n* = 219)	** − 0.429 (0.169)**	**0.011**	** − 0.282 (0.167)**	**0.092**	− 0.254 (0.168)	0.130
Vulnerable depression^7^ (**n** = 50)	** − 1.394 (0.331)**	< 0.001*	** − 1.024 (0.326)**	0.002*	** − 0.997 (0.326)**	0.002*
* p*-trend		**0.018**		**0.027**		**0.048**
In women (*n* = 4639)						
Reference^4^ (*n* = 1697)	0.00 (ref)		0.00 (ref)		0.00 (ref)	
Resilient^5^ (*n* = 2264)	**0.368 (0.082)**	< 0.001*	**0.276 (0.079)**	< 0.001*	**0.280 (0.079)**	< 0.001*
Reactive depression^6^ (*n* = 516)	− 0.191 (0.128)	0.135	0.003 (0.125)	0.981	0.021 (0.125)	0.864
Vulnerable depression^7^ (*n* = 162)	− 0.407 (0.208)	0.051	0.224 (0.203)	0.269	− 0.199 (0.203)	0.326
* p*-trend		0.890		0.680		0.572

^*^Statistically significant after Bonferroni correction for multiple testing

^1^Adjusted for age and study center

^2^Adjusted for age, study center, education, income, marital status, comorbidity, and menopausal status (in women only)

^3^Adjusted for age, study center, education, income, marital status, comorbidity, menopausal status (in women only), alcohol consumption, cigarette smoking, and physical activity

^4^No negative event in 6 months and no current depressive symptoms (BDI < 20)

^5^Experienced negative events in 6 months but no current depressive symptoms (BDI < 20)

^6^Experienced negative events in 6 months with current depressive symptoms (BDI ≥ 20)

^7^No negative event in 6 months and with current depressive symptoms (BDI ≥ 20)

**Table 3:** Association between resilience status and cognitive function stratified by inflammatory biomarkers (*n* = 5327)

Resilience status	Low hsCRP (from the lowest value to 75^th^ %tile)				High hsCRP (from 75^th^ %tile to the highest value)			
	*n*	MMSE mean ± SD	*β*^1^ (SE)	*p*-value	*n*	MMSE mean ± SD	*β*^*1*^ (SE)	*p*-value
In men (*p*-int = 0.587)								
Reference^2^	528	26.67 ± 2.36	Reference		181	26.36 ± 2.63	Reference	
Resilient^3^	665	26.62 ± 2.43	− 0.026 (0.122)	0.832	209	26.61 ± 2.99	0.060 (0.258)	0.815
Reactive depression^4^	90	26.00 ± 2.92	− 0.239 (0.241)	0.323	41	25.95 ± 2.38	− 0.322 (0.438)	0.463
Vulnerable depression^5^	21	24.95 ± 3.01	− **0.970** **(0.465)**	**0.037**	7	25.57 ± 2.76	− 0.515 (0.960)	0.592
*p*-trend				0.165				0.789
In women (*p*-int = 0.306)								
Reference^2^	913	26.09 ± 2.75	Reference		333	26.17 ± 2.60	Reference	
Resilient^3^	1382	26.41 ± 2.56	**0.276** **(0.096)**	0.004^*^	450	26.46 ± 2.48	0.035 (0.159)	0.827
Reactive depression^4^	287	25.64 ± 2.98	0.084 (0.154)	0.585	101	25.35 ± 2.99	− 0.267 (0.253)	0.291
Vulnerable depression^5^	88	25.00 ± 2.81	− 0.399 (0.251)	0.113	31	25.16 ± 3.29	− 0.262 (0.415)	0.528
*p*-trend				0.004				0.603

*MMSE *Mini-mental health examination

^*^Statistically significant after Bonferroni correction for multiple testing

^1^Adjusted for age, study center, education, income, marital status, comorbidity, menopausal status (in women only), alcohol consumption, cigarette smoking, and physical activity

^2^No negative event in 6 months and no current depressive symptoms (BDI < 20)

^3^Experienced negative events in 6 months but no current depressive symptoms (BDI < 20)

^4^Experienced negative events in 6 months with current depressive symptoms (BDI ≥ 20)

^5^No negative event in 6 months and with current depressive symptoms (BDI ≥ 20)

## Discussion

In this study, the resilient group showed higher global cognitive function, and this was more prominent and statistically significant in women. In a fully adjusted model, the vulnerable depression group showed significantly lower global cognitive function compared to the reactive depression group, especially in men. Inflammation status measured with hsCRP seemed to modify these relationships; the pattern was different with respect to sex. Compared to the reference group, being resilient had a higher association with cognitive function in the lower inflammatory group only.

In our data, the reactive depression group showed a higher average number of stressful life events compared to the resilient group. This may imply that people who experience more than one stressful life event on average are more likely to fall into the reactive depression group, and in part explain the lack of resilience.

It is difficult to draw a conclusion regarding the temporal sequence between resilience and global cognitive function, since this study was conducted in a cross-sectional manner. Several review papers suggested that resilience and high cognitive functioning occur simultaneously [27]; whereas, other studies asserted that cognitive impairments may affect the process of adaptive emotional regulation, which is closely related with psychological resilience [28]. As for the prospective study evaluating the impact of resilience on cognitive function, a North American longitudinal study [11] examined the influence of resilience on the association between amyloid-β and cognitive decline, suggesting that people with lower resilience exhibited stronger tendency of amyloid-related cognitive decline. However, education level was the only variable reflecting resilience in this study.

More comprehensive measurements of resilience were used in two cross-sectional studies, evaluating the relationship between resilience and cognitive function related domains. A study [13] conducted in the USA operationally defined resilience with trauma (measured with the Childhood Trauma Questionnaire and Traumatic Events Inventory), depression (measured with the Beck Depression Inventory), and the Post-traumatic Stress Disorder Symptom Scale. The study consisted of 226 highly traumatized low socio-economic civilians, and resilience was significantly associated with nonverbal memory. The population of this study was comparable with our study sample in terms of urban residence and age; however, the resilient group was compared to the group with depression after trauma, whereas our referent group consisted of people without depression or trauma experience. In our analyses, we were able to demonstrate a detailed comparison among the resilient group (i.e., reactive depression group and vulnerable depression group), since we had a much larger population. A study conducted in young Chinese population (mean age 22–23 years) [12], using the Connor-Davidson Resilience Scale to capture psychological resilience, compared 115 mentally ill patients and 52 healthy controls. A significant correlation between resilience and cognitive measures was detected only in the healthy population, which was in line with our results. Our analyses also contained a relatively healthy population; the population from our study reported a mean BDI-II score of 10.6, which was a relatively low score compared to the previous measurement in Korean elderly population studies [29].

Regarding the sex difference in our results, differential reporting between men and women should be considered. In a previous study using the functional MRI to test emotion reactivity regulation to the same negative stimuli, men and women showed different patterns of activation of certain brain area, such as amygdala or pre-frontal area [30]. This may indicate that men could be less likely to perceive “negative” life events, or exert less effort in using cognitive regulation.

Elevated basal level of specific inflammatory markers, including CRP, IL-6, and IL-1, are known to be associated with depressive symptoms [31]. A shared inflammatory etiology, stemming from a cytokine-induced imbalance in the kynurenine pathway, was suggested for both depression and cognitive impairment [32]. Pro-inflammatory cytokines are known to influence the hypothalamic–pituitary–adrenal (HPA) axis [33] and directly affect the nervous system via various routes [33]. Our results imply that inflammation measured as hsCRP might differently influence the relationship between resilience and global cognitive function. Acute inflammation may affect cognition at a much higher level than the protective influence of psychological resilience, or inflammatory cytokines may influence depressive symptoms and cognitive function differently. In our study population, the cutoff for men for the 75^th^ percentile hsCRP was 1.39 mg/L and higher; and for women, higher 75^th^ percentile hsCRP was 1.10 mg/L and higher. In our sensitivity analysis with the cutoff of 3.00 mg/L, since the known normal range for hsCRP is 0.00–3.00 mg/L [24, 25], we observed a similar result compared to our main analysis. Approximately the top 10% of our study population showed hsCRP higher than 3.0 mg/L, which may imply some pathological process behind the scene; our higher hsCRP strata may reflect people with chronic comorbidity, and this may affect cognitive decline stronger than resilience status can protect. Additionally, as CRP is known to be correlated with the amount of adipose tissue [34], it is possible that the association between resilience and cognitive function may be moderated by the body composition.

However, we did not observe any significant interaction between resilience status and IL-6 level on MMSE scores. Compared to people who had measured hsCRP values (2896 men and 4639 women), we had a relatively small number of participants who had valid IL-6 values (217 men and 512 women), which might make it difficult to observe a statistically significant difference. We were still able to observe different patterns by IL-6 status; and in condition with lower IL-6 level, men with reactive depression showed decreased MMSE. However, further study with sufficiently powered population is needed.

This study had several methodological limitations. First, the definition of psychological resilience in our study might not be identical with previous studies, and we focused the relative resilience instead of other potential measurements, such as the Connor-Davidson Resilience Scale [35] or heart rate variability [36]. We could not utilize the information regarding positive adjustments, such as optimism; and yet, more current definitions of resilience include low psychopathology and high positive adjustment. Additionally, we could not obtain information regarding childhood trauma, which is known to play a substantial role in developing resilience. Second, as mentioned previously, the cross-sectional nature of this study limited the causal interpretation of the results. Although depression was measured with psychometrics and global cognitive function was measured objectively, a recall bias may still exist, indicating the possibility of cognitive decline affecting the depressive moods in measuring negative life events. Also, different time frames and the possibility of reverse event order (i.e., depression measured first, and a negative event occurred after) in defining psychological resilience may also be limitations of this study. Third, the MMSE cannot assess specific domains of cognition, such as verbal memory or nonverbal reasoning, and it cannot be used for formal dementia diagnosis [37]. However, there is certain evidence that MMSE has sufficient property to detect cognitive impairment in public health settings [38]. Fourth, measurement errors in the exposure, outcome, and moderation variable is possible. For this reason, we conducted several sensitivity analyses and found consistent findings. Fifth, although we controlled for various factors in the final model, residual confounding regarding the socio-economic, environmental, and neurocognitive factors is possible. Also, unmeasured confounding regarding lifetime psychiatric history may be another limitation. Notably, we found the e-value estimate of 1.95 for resilient women, whereas the e-value estimate was 5.35 for men with vulnerable depression associated with MMSE.[39, 40]; it means that observed linear coefficients (*β* =  − 0.997/ 0.280) between resilient men/ vulnerably depressed women and MMSE could be explained away by possible unmeasured confounders above and beyond by a risk ratio of 1.95/5.35-fold each. Lastly, since this study only included people aged 50 years or older, the findings from our analyses cannot be generalized to other age groups.

Nonetheless, the current study shows strength in several aspects. In this study, we utilized a large well-characterized data with extensive information on potential confounding factors, including socio-demographic factors, lifestyle factors, and physical health status. As far as we know, we utilized the information from 7535 participants, which is the largest population used to evaluate the association between psychological resilience and cognition. The sample size of previous studies ranged from 69 to 276 (Supplementary table 4). Using a sufficiently large sample size, we were able to make detailed categories; for example, in measuring depression, we could further categorize “reactive depression” and “vulnerable depression” categories according to the prior experience of negative life event. Also, we could compare the differential association between resilience and cognition by sex, age group, and inflammatory status. Lastly, we performed a number of sensitivity analyses to define the exposure, outcomes, and modulating factors to corroborate the robustness of our findings.

## Conclusions

In summary, we found a significantly positive association between psychological resilience and global cognitive function in a Korean population of middle-aged and older adults; this pattern was prominent with low inflammatory status. As our sample included healthy urban middle-aged and older adults, further analyses must be done in clinical groups with different age groups and additional inflammatory markers should be tested. Also, measuring specific domains of cognition might further explain the relationship between resilience and cognition. However, findings from our study may serve as substantial evidence of intervention for preventing cognitive decline and dementia in the middle-aged and older adults by building psychological resilience in midlife through various community programs, together with controlling chronic inflammation. Overall, our results should be further replicated in other large population longitudinal settings to elucidate temporality and causal relationships.

## Supplementary Information

Below is the link to the electronic supplementary material.

Supplementary file1 (DOCX 54 KB)

## Abbreviations

CMERC: Cardiovascular and Metabolic Diseases Etiology Research Center
LES: Life Experience Survey
hsCRP: High-sensitivity C-reactive protein
MMSE: Mini-mental state examination
HPA: Hypothalamic-pituitary-adrenal (HPA)
OR: Odds ratio
95% CI: 95% Confidence interval
SD: Standard deviation
