# Supplementary material for: Association between psychological resilience and cognitive function in older adults: effect modification by inflammatory status
Source: GeroScience. 2021 Jun 29;43(6):2749–60. doi: 10.1007/s11357-021-00406-1 (PMC8238632; doi:10.1007/s11357-021-00406-1)
Supplement: Supplementary file 1 — Supplementary file1 (DOCX 54 KB) [file 11357_2021_406_MOESM1_ESM.docx]

| Supplementary table 1. Comparison of baseline characteristics between included and excluded populations | | | |
| --- | --- | --- | --- |
| Participant characteristics | Included  (n=7,535) | Excluded  (n=4,429) | p-value |
| Age, mean (SD) | 58.9 (6.2) | 45.1 (10.1) | <0.001 |
| Female, n (%) | 4,635 (61.6) | 2,470 (55.8) | <0.001 |
| Socio-economic variables |  |  |  |
| Education: high school or higher, n (%) | 2,466 (32.7) | 2,467 (55.7) | <0.001 |
| Highest quartile of yearly household income, n (%) | 1,686 (22.4) | 897 (20.3) | 0.007 |
| Currently married, living together, n (%) | 6,504 (86.3) | 3,601 (81.3) | <0.001 |
| Presence of major comorbidity, n (%) | 5,016 (67.6) | 1,926 (43.7) | <0.001 |
| Hypertension, n (%) | 3,655 (48.5) | 1,553 (35.1) | <0.001 |
| Diabetes, n (%) | 1,736 (23.0) | 549 (12.4) | <0.001 |
| Body mass index (kg/m^2^), mean (SD) | 24.5 (3.1) | 24.3 (3.6) | 0.068 |
| Lifestyle factors, n (%) |  |  |  |
| Current cigarette smoker | 703 (9.3) | 906 (20.5) | <0.001 |
| Current alcohol consumer | 4,578 (60.8) | 3,116 (70.4) | <0.001 |
| Regular exercise^5^ | 2,647 (35.1) | 1,472 (33.3) | 0.037 |
| Menopause (women only) | 4,241 (91.4) | 576 (23.3) | <0.001 |
| Psychiatric assessments |  |  |  |
| Beck Depression Inventory^6^ II (range: 0-63) | 10.6 (7.8) | 10.0 (7.2) | <0.001 |
| Mini Mental State Examination-DS (range: 0-30) | 26.5 (2.7) | 26.5 (3.9) | 0.823 |
| ^1^No negative event experience in 6 months and no current depressive symptoms (BDI<20) | | | |
| ^2^Experienced negative events in 6 months but no current depressive symptoms (BDI<20) | | | |
| ^3^Experienced negative events in 6 months with current depressive symptoms (BDI≥20) | | | |
| ^4^No negative event experience in 6 months and with current depressive symptoms (BDI≥20) | | | |
| ^5^Defined as having moderate-vigorous physical activity of more than 150 minutes a week on average | | | |

| Supplementary table 2. Association between psychologic resilience status and cognitive decline categorized by MMSE-DS cutoff | | | | |
| --- | --- | --- | --- | --- |
| Resilience status | Cognitive decline^1^ by MMSE-DS^2^ cutoff | | | |
|  | Case n (%) | OR^3^ (95% CI) | OR^4^ (95% CI) | OR^5^ (95% CI) |
| In men |  |  |  |  |
| Reference^6^ (n=1,181) | 245 (20.8) | 1.00 (ref) | 1.00 (ref) | 1.00 (ref) |
| Resilient^7^ (n=1,446) | 288 (19.9) | 0.93 (0.76-1.45) | 0.96 (0.78-1.19) | 0.96 (0.78-1.19) |
| Reactive depression^8^ (n=219) | 61 (28.0) | **1.43 (1.00-2.04)** | 1.32 (0.92-1.91) | 1.25 (0.86-1.81) |
| Vulnerable depression^9^ (n=50) | 23 (46.0) | **3.24 (1.73-6.09)** | **2.81 (1.48-5.35)** | **2.70 (1.41-5.16)** |
| In women |  |  |  |  |
| Reference^6^ (n=1,697) | 503 (29.6) | 1.00 (ref) | 1.00 (ref) | 1.00 (ref) |
| Resilient^7^ (n=2,264) | 586 (25.9) | **0.78 (0.67-0.91)** | **0.80 (0.68-0.94)** | **0.80 (0.68-0.93)** |
| Reactive depression^8^ (n=516) | 169 (32.8) | 0.99 (0.78-1.25) | 0.92 (0.73-1.18) | 0.91 (0.72-1.16) |
| Vulnerable depression^9^ (n=162) | 64 (39.5) | 1.26 (0.87-1.81) | 1.22 (0.84-1.78) | 1.20 (0.82-1.75) |
| ^1^Cognitive decline was defined by +1.5SD of each age and educational level strata, provided by Han JW *et al.* J Korean Geriatric Psychiatry 2010;14:24-37. | | | | |
| ^2^Mini-Mental State Examination-Dementia Screening, developed by Han JW *et al.* | | | | |
| ^3^Adjsted for age and study center | | | | |
| ^4^Adjusted for age, study center, education, income, marital status, comorbidity, and menopausal status (in women only) | | | | |
| ^5^Adjusted for age, study center, education, income, marital status, comorbidity, menopausal status (in women only), alcohol consumption, cigarette smoking, and physical activity | | | | |
| ^6^No negative event in 6 months and no current depressive symptoms (BDI<20) | | | | |
| ^7^Experienced negative events in 6 months but no current depressive symptoms (BDI<20) | | | | |
| ^8^Experienced negative events in 6 months with current depressive symptoms (BDI≥20) | | | | |
| ^9^No negative event in 6 months and with current depressive symptoms (BDI≥20) | | | | |

| Supplementary table 3. Sensitivity analysis of resilience status and cognitive function with new resilience definition ^4,5,6,7^ (N=7,535) | | | | | | |
| --- | --- | --- | --- | --- | --- | --- |
| Resilience status | Mini Mental State Examination-Dementia Screening (cont.) | | | | | |
|  | β^1^ (SE) | p-value | β^2^ (SE) | p-value | β^3^ (SE) | p-value |
| In men |  |  |  |  |  |  |
| Reference^4^ (n=1,802) | 0.00 (ref) |  | 0.00 (ref) |  | 0.00 (ref) |  |
| Resilient^5^ (n=825) | 0.183 (0.097) | 0.058 | **0.193 (0.095)** | **0.042** | **0.190 (0.095)** | **0.045** |
| Reactive depression^6^ (n=170) | -0.36 (0.184) | 0.051 | -0.273 (0.181) | 0.132 | -0.236 (0.181) | 0.192 |
| Vulnerable depression^7^ (n=99) | **-1.11 (0.237)** | **<.0001** | **-0.715 (0.236)** | **0.003** | **-0.703 (0.236)** | **0.003** |
| In women |  |  |  |  |  |  |
| Reference^4^ (n=2,618) | 0.00 (ref) |  | 0.00 (ref) |  | 0.00 (ref) |  |
| Resilient^5^ (n=1,343) | **0.388 (0.086)** | **<.0001** | **0.280 (0.083)** | **0.001** | **0.279 (0.083)** | **0.001** |
| Reactive depression^6^ (n=386) | -0.228 (0.138) | 0.100 | -0.053 (0.135) | 0.693 | -0.037 (0.135) | 0.783 |
| Vulnerable depression^7^ (n=292) | **-0.444 (0.156)** | **0.005** | -0.195 (0.153) | 0.203 | -0.176 (0.153) | 0.251 |
| ^1^Adjsted for age and study center | | | | | | |
| ^2^Adjusted for age, study center, education, income, marital status, comorbidity, and menopausal status (in women only) | | | | | | |
| ^3^Adjusted for age, study center, education, income, marital status, comorbidity, menopausal status (in women only), alcohol consumption, cigarette smoking, and physical activity | | | | | | |
| ^4^Less than two negative events in 6 months and no current depressive symptoms (BDI<20) | | | | | | |
| ^5^Experienced more than two negative events in 6 months but no current depressive symptoms (BDI<20) | | | | | | |
| ^6^Experienced more than two negative events in 6 months with current depressive symptoms (BDI≥20) | | | | | | |
| ^7^Less than two negative events in 6 months and with current depressive symptoms (BDI≥20) | | | | | | |

| \| Supplementary table 4. Demographic characteristics of studies examining psychological resilience and cognition \| \| \| \| \| \| \| \| \| --- \| --- \| --- \| --- \| --- \| --- \| --- \| --- \| \| **Authors** \| **Publication year** \| **Study design** \| **Participants** \| **Participants (N)** \| **Mean age (years)** \| **Resilience definition** \| **Cognition measurement** \| \| Wingo *et al*. \| 2010 \| Cross-sectional \| US, highly traumatized civilians \| 226 \| 44-45 \| Operational definition from the Childhood Trauma Questionnaire, Traumatic Events Inventory, Beck Depression Inventory, and Posttraumatic Stress Disorder Symptom Scale \| Reynolds Intellectual Assessment Scales \| \| Wolf *et al.* \| 2019 \| Longitudinal, with 10 year follow-up \| US and Canada, ADNI database, cognitively healthy at baseline \| 276 \| 74.4 \| Education level \| Alzheimer Disease Assessment Scale-13-item cognitive subscale, Clinical Dementia Rating-Sum of Boxes, Alzheimer's Disease Neuroimaging Initiative -memory composite score \| \| Deng *et al.* \| 2018 \| Cross-sectional \| China, HC(52)+Schizo(81)+BP(34) \| 167 \| 22-23 \| Connor Davidson Resilience Scale \| Wechsler Adult Intelligence Scale-Chinese Revised, Verbal Fluency(executive functioning)+N-back task (working memory) \| \| Hoorelbeke *et al.* \| 2016 \| Cross-sectional \| Belgium, 69 remitted depression patients \| 69 \| 47.13 \| Resilience Scale \| Paced Auditory Serial Addition task (behavioral indicator of cognitive control) \| |
| --- | --- | --- | --- | --- | --- | --- | --- | --- | --- | --- | --- | --- | --- | --- | --- | --- | --- | --- | --- | --- | --- | --- | --- | --- | --- | --- | --- | --- | --- | --- | --- | --- | --- | --- | --- | --- | --- | --- | --- | --- | --- | --- | --- | --- | --- | --- | --- | --- |

Supplementary box 1.

List of interpersonal adversities selected from the Life Experience Survey

• Death of spouse

• Death of one’s mother

• Death of one’s father

• Death of one’s siblings

• Death of one’s grandmother

• Death of one’s grandfather

• Death of one’s close friend

• Having conflict with one’s manager

• Having conflict with relatives

• Having conflict with family members

• Experience of reconciliation with spouse

• Experience of a change in the number of quarrels with one’s spouse

• Experiencing changes in social activities, such as meetings or visits

• Having divorced

• Experiencing a breakup with a friend of the opposite sex

• Experiencing reconciliation with a friend of the opposite sex

Supplementary table 5. Number of stressful life events in recent 6 months by resilience status

|  | Reference | | Resilient | | Reactive depression | | Vulnerable depression | |
| --- | --- | --- | --- | --- | --- | --- | --- | --- |
|  | Total, n | NoSLE, mean+SD | Total, n | NoSLE, mean+SD | Total, n | NoSLE, mean+SD | Total, n | NoSLE, mean+SD |
| Men | 1,181 | 1.1+1.6 | 1,446 | 4.0+3.0 | 219 | 5.1+3.9 | 50 | 1.2+1.5 |
| Women | 1,697 | 1.0+1.5 | 2,264 | 4.1+2.9 | 516 | 4.8+3.2 | 162 | 0.9+1.5 |

Abbreviation: NoSLE= Number of Stressful Life Event

| Supplementary table 6. Association between resilience status and cognitive function stratified by hsCRP (n=5,327) | | | | | | | | | | | | | | | | |
| --- | --- | --- | --- | --- | --- | --- | --- | --- | --- | --- | --- | --- | --- | --- | --- | --- |
| Resilience status | Low hsCRP ( 0-3 mg/L) | | | | | | |  | High hsCRP Low hsCRP ( 3+ mg/L) | | | | | | | |
|  | n | MMSE mean ± SD | | | β^2^ | (SE) | p-value |  | n | MMSE mean ± SD | | | β^2^ | (SE) | p-value |  |
| In men (p-int=0.587) |  |  |  |  |  |  |  |  |  |  |  |  |  |  |  |  |
| Reference^2^ | 635 | 26.60 | ± | 2.41 | Reference | |  |  | 74 | 26.49 | ± | 2.66 | Reference | |  |  |
| Resilient^3^ | 800 | 26.61 | ± | 2.58 | -0.020 | (0.117) | 0.861 |  | 74 | 26.72 | ± | 2.44 | 0.288 | (0.396) | 0.468 |  |
| Reactive depression^4^ | 117 | 26.09 | ± | 2.73 | -0.183 | (0.222) | 0.409 |  | 14 | 25.14 | ± | 2.85 | -0.760 | (0.700) | 0.279 |  |
| Vulnerable depression^5^ | 24 | 24.96 | ± | 3.09 | **-0.986** | **(0.453)** | **0.030** |  | 4 | 26.00 | ± | 1.41 | 0.141 | (1.233) | 0.909 |  |
| p-trend |  |  |  |  |  |  | 0.155 |  |  |  |  |  |  |  | 0.515 |  |
| In women (p-int=0.306) |  |  |  |  |  |  |  |  |  |  |  |  |  |  |  |  |
| Reference^2^ | 1148 | 26.14 | ± | 2.70 | Reference | |  |  | 98 | 25.78 | ± | 2.72 | Reference | |  |  |
| Resilient^3^ | 1710 | 26.41 | ± | 2.54 | **0.196** | **(0.086)** | **0.022** |  | 122 | 26.65 | ± | 2.45 | 0.297 | (0.310) | 0.339 |  |
| Reactive depression^4^ | 348 | 25.61 | ± | 3.03 | -0.003 | (0.138) | 0.980 |  | 40 | 25.13 | ± | 2.55 | 0.010 | (0.439) | 0.982 |  |
| Vulnerable depression^5^ | 110 | 25.07 | ± | 2.80 | -0.349 | (0.224) | 0.118 |  | 9 | 24.67 | ± | 4.42 | -1.094 | (0.852) | 0.200 |  |
| p-trend |  |  |  |  |  |  | **0.016** |  |  |  |  |  |  |  | 0.376 |  |
| ^1^Adjusted for age, study center, education, income, marital status, comorbidity, menopausal status (in women only), alcohol consumption, cigarette smoking, and physical activity | | | | | | | | | | | | | | | | |
| ^2^No negative event in 6 months and no current depressive symptoms (BDI<20) | | | | | | | | | | | | | | | | |
| ^3^Experienced negative events in 6 months but no current depressive symptoms (BDI<20) | | | | | | | | | | | | | | | | |
| ^4^Experienced negative events in 6 months with current depressive symptoms (BDI≥20) | | | | | | | | | | | | | | | | |
| ^5^No negative event in 6 months and with current depressive symptoms (BDI≥20) | | | | | | | | | | | | | | | | |

| Supplementary table 7. Association between resilience status and cognitive function stratified by IL-6 level (n=729) | | | | | | | | | | | | | | | | |
| --- | --- | --- | --- | --- | --- | --- | --- | --- | --- | --- | --- | --- | --- | --- | --- | --- |
| Resilience status | Low IL-6 (from the lowest value to 75^th^ %tile) | | | | | | |  | High IL-6 (from 75^th^ %tile to the highest value) | | | | | | | |
|  | n | MMSE mean ± SD | | | β^2^ | (SE) | p-value |  | n | MMSE mean ± SD | | | β^2^ | (SE) | p-value |  |
| In men (p-int=0.687) |  |  |  |  |  |  |  |  |  |  |  |  |  |  |  |  |
| Reference^2^ | 73 | 27.30 | ± | 1.76 | Reference | |  |  | 21 | 27.38 | ± | 1.91 | Reference | |  |  |
| Resilient^3^ | 72 | 27.44 | ± | 1.74 | 0.105 | (0.273) | 0.700 |  | 30 | 27.2 | ± | 2.02 | -0.267 | (0.626) | 0.673 |  |
| Reactive depression^4^ | 15 | 25.67 | ± | 2.26 | -1.030 | (0.477) | 0.032 |  | 3 | 27 | ± | 0 | -0.235 | (1.364) | 0.864 |  |
| Vulnerable depression^5^ | 2 | 28.50 | ± | 2.12 | **-0.090** | **(1.193)** | **0.935** |  | 1 | 27.00 | ± | 0 | -0.770 | (2.163) | 0.724 |  |
| p-trend |  |  |  |  |  |  | 0.932 |  |  |  |  |  |  |  | 0.348 |  |
| In women (p-int=0.203) |  |  |  |  |  |  |  |  |  |  |  |  |  |  |  |  |
| Reference^2^ | 135 | 27.09 | ± | 1.93 | Reference | |  |  | 60 | 26.48 | ± | 2.55 | Reference | |  |  |
| Resilient^3^ | 196 | 27.06 | ± | 1.99 | **-0.050** | **(0.209)** | **0.779** |  | 58 | 27.34 | ± | 1.89 | 0.326 | (0.310) | 0.443 |  |
| Reactive depression^4^ | 46 | 26.96 | ± | 1.97 | 0.262 | (0.330) | 0.428 |  | 8 | 25.75 | ± | 2.49 | 0.295 | (0.439) | 0.741 |  |
| Vulnerable depression^5^ | 7 | 25.43 | ± | 0.79 | -0.810 | (0.719) | 0.257 |  | 2 | 28.5 | ± | 0.71 | 3.816 | (0.852) | 0.092 |  |
| p-trend |  |  |  |  |  |  | **0.032** |  |  |  |  |  |  |  | 0.339 |  |
| ^1^Adjusted for age, study center, education, income, marital status, comorbidity, menopausal status (in women only), alcohol consumption, cigarette smoking, and physical activity | | | | | | | | | | | | | | | | |
| ^2^No negative event in 6 months and no current depressive symptoms (BDI<20) | | | | | | | | | | | | | | | | |
| ^3^Experienced negative events in 6 months but no current depressive symptoms (BDI<20) | | | | | | | | | | | | | | | | |
| ^4^Experienced negative events in 6 months with current depressive symptoms (BDI≥20) | | | | | | | | | | | | | | | | |
| ^5^No negative event in 6 months and with current depressive symptoms (BDI≥20) | | | | | | | | | | | | | | | | |
